# Rational drug repositioning guided by an integrated pharmacological network of protein, disease and drug

**DOI:** 10.1186/1752-0509-6-80

**Published:** 2012-07-02

**Authors:** Hee Sook Lee, Taejeong Bae, Ji-Hyun Lee, Dae Gyu Kim, Young Sun Oh, Yeongjun Jang, Ji-Tea Kim, Jong-Jun Lee, Alessio Innocenti, Claudiu T Supuran, Luonan Chen, Kyoohyoung Rho, Sunghoon Kim

**Affiliations:** 1 Medicinal Bioconvergence Research Center, College of Pharmacy, Seoul National University, Seoul, Korea; 2 World Class University Program Department of Molecular Medicine and Biopharmaceutical Sciences, Seoul National University, Seoul, 151-742, Korea; 3 Information Center for Bio-pharmacological Network, Seoul National University, Suwon, Korea; 4 Dipartimento di Chimica Laboratorio di Chimica Bioinorganica, University of Florence, Via della Lastruccia, 3, Rm. 188 Polo Scientifico, Sesto Fiorentino (Firenze), 50019, Italy; 5 Key Laboratory of Systems Biology, Shanghai Institutes for Biological Sciences, Chinese Academy of Sciences, Shanghai, 200233, China; 6 Medicinal Bioconvergence Research Center, Advanced Institutes of Convergence Technology, Suwon, 443-270, Korea

**Keywords:** Tripartite network, Drug repositioning, Shared Neighborhood Scoring (SNS) algorithm

## Abstract

**Background:**

The process of drug discovery and development is time-consuming and costly, and the probability of success is low. Therefore, there is rising interest in repositioning existing drugs for new medical indications. When successful, this process reduces the risk of failure and costs associated with *de novo* drug development. However, in many cases, new indications of existing drugs have been found serendipitously. Thus there is a clear need for establishment of rational methods for drug repositioning.

**Results:**

In this study, we have established a database we call “PharmDB” which integrates data associated with disease indications, drug development, and associated proteins, and known interactions extracted from various established databases. To explore linkages of known drugs to diseases of interest from within PharmDB, we designed the Shared Neighborhood Scoring (SNS) algorithm. And to facilitate exploration of tripartite (Drug-Protein-Disease) network, we developed a graphical data visualization software program called phExplorer, which allows us to browse PharmDB data in an interactive and dynamic manner. We validated this knowledge-based tool kit, by identifying a potential application of a hypertension drug, benzthiazide (TBZT), to induce lung cancer cell death.

**Conclusions:**

By combining PharmDB, an integrated tripartite database, with Shared Neighborhood Scoring (SNS) algorithm, we developed a knowledge platform to rationally identify new indications for known FDA approved drugs, which can be customized to specific projects using manual curation. The data in PharmDB is open access and can be easily explored with phExplorer and accessed via BioMart web service (http://www.i-pharm.org/, http://biomart.i-pharm.org/).

## Background

Modern drug discovery is time-consuming and expensive, involving coordinated multi-disciplinary research in multiple stages, each requiring intensive and specialized resources [1]. Although rapid advancement of “omics” approaches, computational systems biology and accumulation of digital data resources have provided a vast array of significant information in life science [2], data relevant to drug discovery are not easily identified and recruited for application to pharmaceutical research [3]. Despite the technological advances in drug discovery such as HTS, the approval of new drugs has remained stagnant in the past decade, resulting in an overall decline in the productivity of` the pharmaceutical industry.

In efforts to save development time and minimize the risk of failure during drug develo pment, repositioning of currently available drugs to new therapeutic indications is considered an alternative route [1]. To date most repositioned drugs have been the consequence of serendipitous observations of unexpected efficacy and side effects of drugs in development or on the market. However, recently, systems biology approaches have been applied in efforts to discover unknown effects for existing drugs. For instance, drug repositioning approaches have incorporated *in silico* approaches for analyzing large data sets such as gene expression profiles [4,5], literature mining [6], chemical similarity [7], side-effect similarity [8], disease-drug network [9], pathway-based disease network [10], and phenotypic disease network [11]. To establish a more logical approach to repositioning a known drug to a new indication, we established a knowledge platform comprising binary linkages between diseases, drugs, and proteins, from which new and previously unknown connections can be drawn between drugs and diseases of interest. This integrated database was designated PharmDB.

For probing the database and identifying disease-drug linkages, we have developed the Shared Neighborhood Scoring (SNS) algorithm, which predicts relationships between drugs, proteins and diseases. While the relationship data are collected from experiments, coverage of the data is still incomplete. Thus there may be undetected links and hidden nodes in the network. Up to now, a number of prediction methods and measures have been proposed to find these undetected associations from topological or structural properties of various complex networks [12,13]. To date, most of these algorithms and measures are applicable only to a monopartite network that consists only of one type of node. Therefore, multipartite network composed of more than a type of nodes cannot be analyzed using these measures. To solve this problem, researchers have used projection methods that convert multipartite networks into monopartite ones. Unfortunately, any projection method can result in information loss, especially in low-degree nodes. Accordingly projecting the PharmDB tripartite network into monopartite drug, protein and disease networks can distort many well-known network measures, such as average path length < l>, average clustering coefficient < C>, degree-dependent clustering coefficient C(k), degree distribution P(k), assortativity coefficient r [14], and degree-degree correlation coefficient k_nn_(k) [15]. To overcome these limits of the projection technique, we designed a new prediction method called Shared Neighborhood Scoring (SNS) algorithm which calculates the probability of a link existence between two nodes of interest. This can be done by evaluating the connections of their neighbors in PharmDB tripartite network.

## Results

### System overview

The PharmDB is a tripartite pharmacological network database consisting of three kinds of nodes: human diseases, FDA approved drugs or druggable chemicals, and proteins. The proteins in PharmDB include therapeutic targets, disease-associated proteins, and drug-metabolizing proteins. The nodes and links used to construct this network database were imported from nine public databases, namely, EntrezGene interaction [16], MINT [17], DIP [18], CTD [19], TTD [20], ChemBank [21], PharmGKB [22], OMIM [23], and GAD [24] (Table 1).

**Table 1:** Data sources of PharmDB

	**Drug-Protein Relation**	**Protein-Disease Relation**	**Drug-Disease Relation**	**Protein-Protein Interaction**	**Update Date**
EntrezGene Interaction				V	2011.07.20
MINT				V	2011.07.08
DIP				V	2010.10.10
PharmGKB	V	V	V	V	2011.07.20
CTD	V	V	V		2011.07.11
TTD	V	V	V		2011.07.04
ChemBank			V		2011.07.21
OMIM		V			2011.03.10
GAD		V			2011.07.16

**Figure 1:**
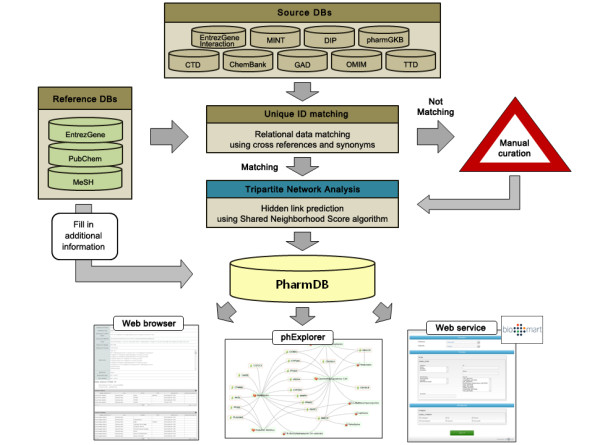
**Overview of PharmDB.** Nine different databases were integrated using standard IDs (Entrez Gene ID for protein, PubChem CID for drug and MeSH Descriptor ID for disease) to construct PharmDB. The integrated network was analyzed using the shared neighborhood scoring algorithm, providing a predictive capacity for PharmDB to suggest functional relationships between diseases, proteins, and rugs. These data are provided through a web browser, phExplorer (network visualization software) and web service (http://www.i-pharm.org/, http://biomart.i-pharm.org/).

Although these individual databases provide information about the relationships between drugs, diseases, and proteins, they do not provide an integrated network map among the three components in an interactive manner. For data integration in a unified format, we adopted PubChem CID for drugs, GeneID for proteins (tagging separate IDs for isozymes and subunits), and MeSH descriptor for diseases (Figure 1). PharmDB currently includes the nodes of 11,792 drugs, 38,056 proteins, and 6,607 diseases. It also contains 189,800 Drug-Protein, 109,124 Protein-Disease, and 12,232 Drug-Disease, 156,902 Protein-Protein links. The contents of the tripartite pharmacological network in PharmDB are provided through a website (http://www.i-pharm.org/). phExplorer, a graphical data visualization software program is also provided for interactive browsing of relevant data. For constructing workflows, PharmDB is provided in BioMart format (http://biomart.i-pharm.org/). Currently, software for finding the shortest path between two nodes is only provided through the website.

### Shared neighborhood scoring (SNS) algorithm

**Figure 2:**
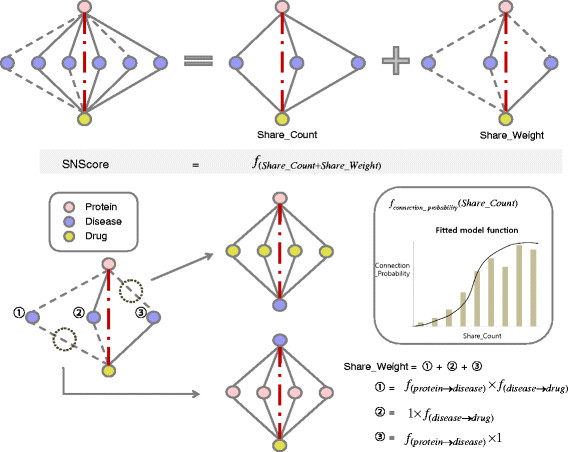
**Shared neighborhood scoring algorithm.** SN score can be calculated by summing up "Shared Nodes Count" and "Shared Nodes Weight". First, “Shared Nodes Count” is defined as the number of shared nodes to consider the effect of direct connectors. Similary, “Shared Nodes Weight” is defined as the product of each weight of links bridging two end nodes to trace the effect of indirect neighbors (Bottom right). Here weight is a measure for connecting probability of each pair. For all possible pairs of the network, firstly weight 1 is assigned to each connected pairs directly linked between two nodes. Weight for unconnected pairs is assigned the connection probability, the fraction of directly connected pairs among the total number of pairs having the given “Shared Nodes Count”. For example, (1) the weight product of two indirect links (2), (3) weight of the upper (direct link / indirect link) multiplied by weight of the lower one (indirect link / direct link). The sum of (1), (2), and (3) is "Shared Weight" of the unconnected pair (protein, drug) (bottom left).

The concept of SNS algorithm is similar to Swanson’s ABC model, which applies the transitivity rule to discover missing knowledge from biomedical literature [25]. The SNS algorithm is based on the observation that the probability of connection between two nodes shows monotonic increase with “Shared Nodes Count”, the number of in-between nodes connecting two nodes (Figure 2, middle left box). Further we calculated weights for all possible pairs of the network. First we assigned weight 1 to each connected pairs directly linked between two nodes. If a pair of two nodes is not connected, the connection probability is assigned as weight for this indirect link or a virtual link between two nodes. As shown in the Figure 2, the connection probability for given “Shared Nodes Count” can be computed to be the fraction of directly connected pairs among the total number of pairs having the given “Shared Nodes Count”. Finally we developed the share neighborhood score (SN score) by summing up “Shared Nodes Count”, the number of shared nodes and “Shared Nodes Weight”, the product of each weight of (direct or indirect) links bridging the two end nodes (Figure 2, bottom left). As the SN score possesses a range of values in each relation category (drug-protein, protein-disease, and drug-disease), we developed a normalization method using the connecting probability function of SN score distribution (see Materials and Methods for details).

**Figure 3:**
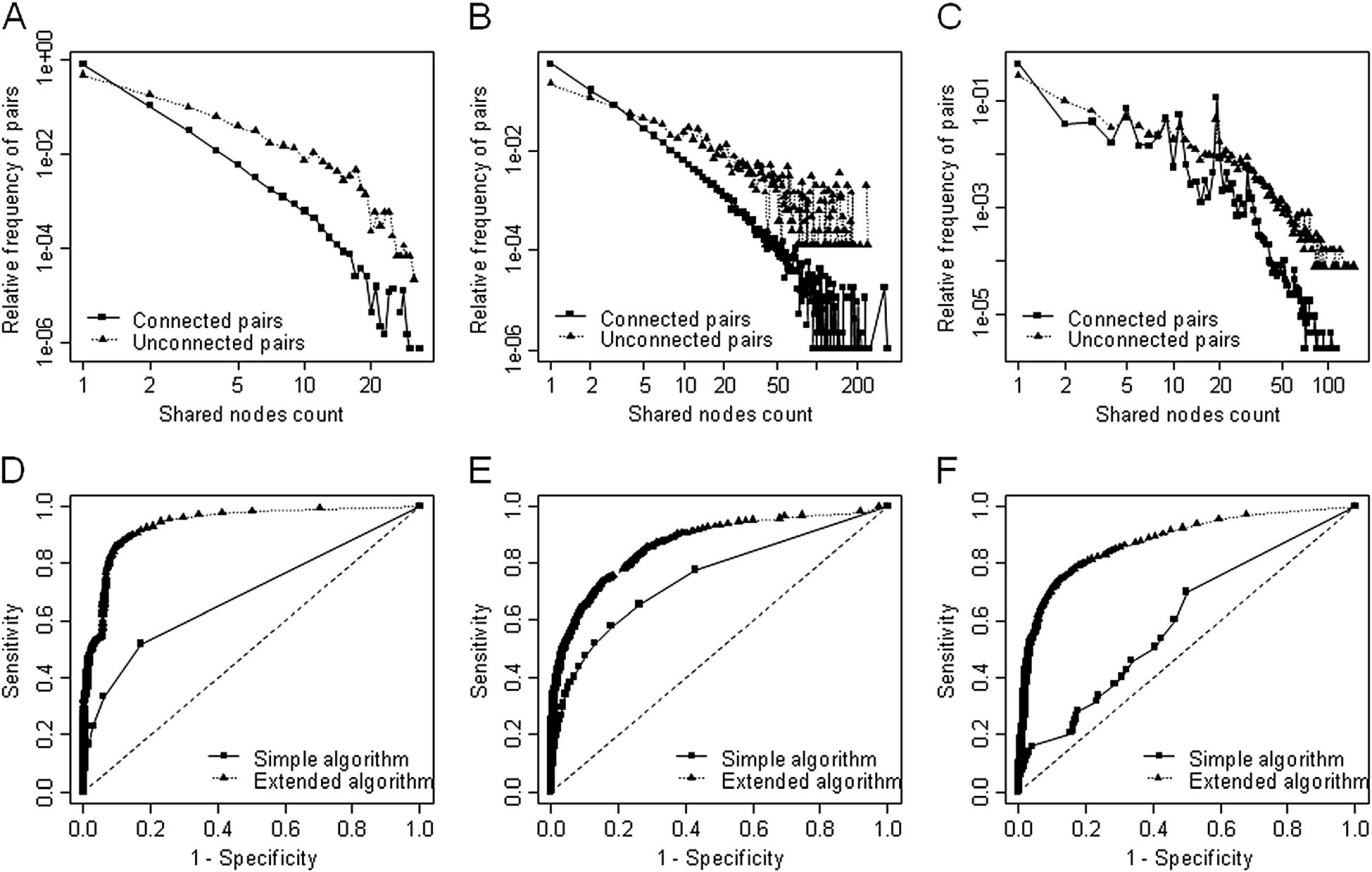
**Shared neighborhood node distribution and evaluation of the shared neighborhood scoring algorithm.** Shared neighborhood node distribution comparison between connected links and unconnected links in Drug-Protein relation ( **A**), Drug-Disease relation ( **B**) and Protein-Disease relation ( **C**) (Rectangle: Connected links, Triangle: Unconnected links). ROC analysis of simple form of SNS algorithm and extended form of SNS algorithm in Drug-Protein relation ( **D**), Drug-Disease relation ( **E**) and Protein-Disease relation (F) (Rectangle: Simple algorithm, Triangle: Extended algorithm).

We compared the “Shared Nodes Count” distribution for connected pairs and unconnected pairs. Connected pairs shared more neighborhood nodes than unconnected pairs. The p-values of the Kolmogorov-Smirnov (KS) test are less than 2.2e-16 in all three relation categories, meaning that connected pairs and unconnected pairs have significantly distinct distribution (Figure 3A, 3B, 3 C).

The prediction performance of the SNS algorithm was measured by plotting receiver operating characteristic (ROC) curves (Figure 3D, 3E, 3 F). For calculating SN scores, “simple algorithm” considers only “Shared Nodes Count” but “extended algorithm” includes both “Shared Nodes Count” and “Shared Nodes Weight”. As shown in the Figure 3, the extended algorithm shows better performance than simple one. AUC values of simple algorithm are 0.679, 0.778, and 0.602, in Drug-Protein relation, Drug-Disease relation, and Protein-Disease relation, respectively. And AUC values for extended algorithm are 0.937, 0.868, and 0.871. According to the result, prediction performances with extended scope of shared neighborhood nodes were improved by 38%, 12%, and 45%, respectively.

### Case study – benzthiazide as a potential agent for lung cancer

**Figure 4:**
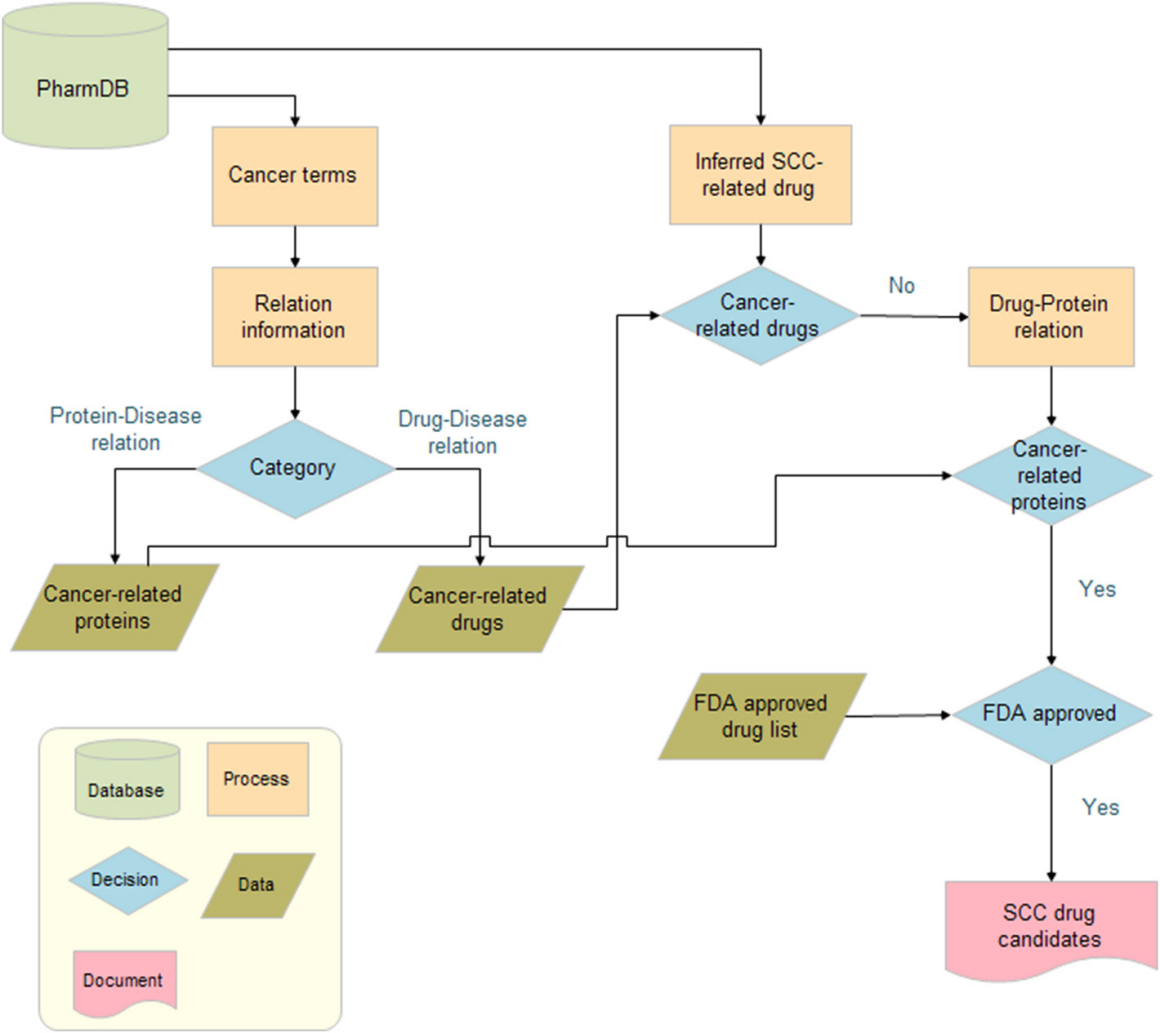
**Drug repositioning pipeline overview.** Schematic representation of drug repositioning pipeline for squamous cell carcinoma (SCC). First, cancer-related proteins and drugs were extracted from PharmDB using cancer terms (such as “Carcinoma”, “Neoplasm”, and “Cancer”). Second, inferred SCC-related drugs were extracted using the shared neighborhood scoring algorithm. Among the candidates, any known cancer agents were filtered out; leaving only drugs that had not been previously implicated as anti-cancer drugs. Then the FDA approved drugs which known to be related with cancer-related proteins were maintained for further analysis as SCC drug candidates in this study.

**Figure 5:**
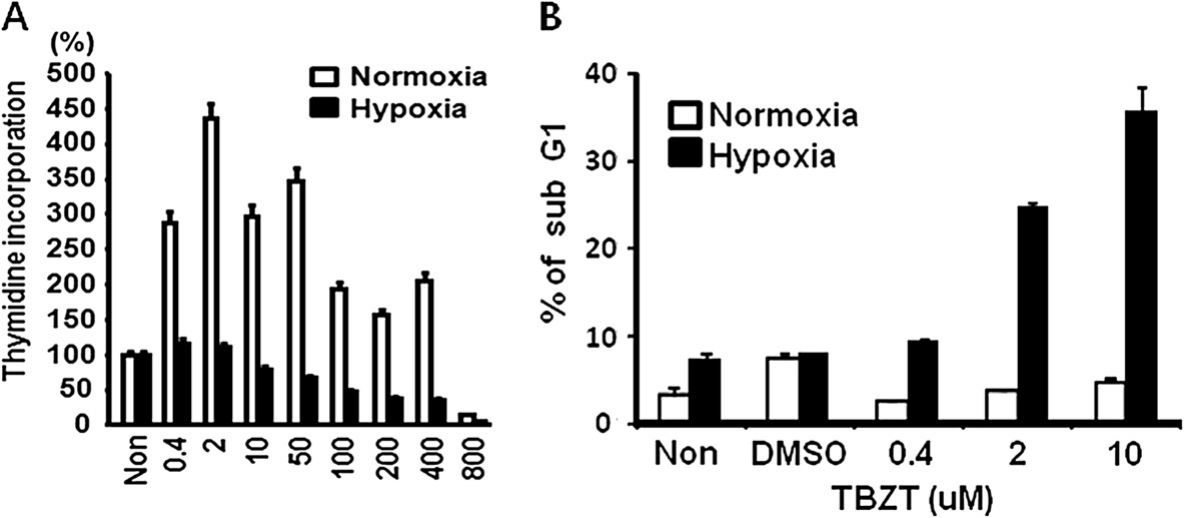
**The hypoxia-dependent TBZT effect against SCC.** ( **A**) Antiproliferative activity of TBZT was monitored by [^3^ H] thymidine incorporation under normoxic and hypoxic conditions. (**B**) The effect of TBZT on cell death was monitored by counting sub-G1 cells.

As a case study, we chose squamous cell carcinoma (SCC) (MeSH descriptor: D002294), a subtype of lung cancer, and tested whether PharmDB could identify any drugs that have a potential for treating this type of cancer. For the primary selection of drug candidates in this case, we made the following criteria. First, they should be inferred by SNS algorithm with SN score bigger than 0.004 and Share Nodes Count zero. Second, they should belong to FDA approved drugs. Third, they should not have been previously used for cancer drug. Forth, they should be directly linked to cancer target proteins (Figure 4). Twenty eight common drugs fit to the four criteria above and were suggested as potential SCC drug candidates (Additional file 1: Table S3). We then went over these candidates to choose the one for experimental validation. Considering technical feasibility, availability of materials, intellectual property and potential for new drug development, we decided to examine thia-benzthiazide (TBZT) whether it can be used for SCC treatment. TBZT is a kind of thiazide diurectic used for the treatment of high blood pressure and edema [26]. To validate a potential of TBZT as a lung cancer drug, we administered different concentrations of TBZT to squamous lung cancer cells (HCC-1588) under hypoxic conditions (which mimic the tumor microenvironment), as well as under normoxic conditions [27]. Their effects on cell proliferation were monitored by [^3^ H] thymidine incorporation. We found that under hypoxic conditions only, TBZT can suppress proliferation of cancer cell in a dose-dependent manner (Figure 5A). The hypoxia-dependent cell death induced by TBZT was further confirmed by flow cytometry (Figure 5B).

**Figure 6:**
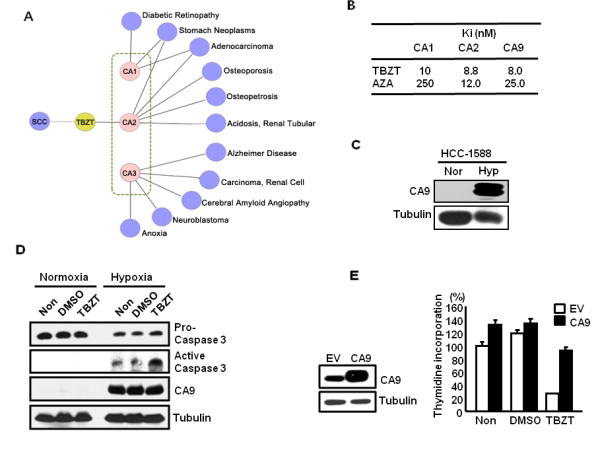
**TBZT as an inhibitor of CA9.** ( **A**) To validate predictions by PharmDB analysis for SCC, TBZT is tested for inhibitory activity against its potential targets, CA isozymes (CA1, CA2, and CA9). ( **B**) *In vitro* inhibition of TBZT and the AZA control against CA isoforms (i.e., 1, 2, and 9). ( **C**) Cellular levels of CA9 in the SCC cell line, HCC-1588, under normoxic and hypoxic conditions. ( **D**) The effect of TBZT on cell death was monitored by caspase-3 activation. (E) HCC-1588 cells, transfected with an empty vector (EV) or CA9, were treated with TBZT under normoxic and hypoxic conditions.

Carbonic anhydrases (CAs) are zinc metalloenzymes which catalyze the conversion of carbon dioxide to the bicarbonate ion and protons. The CAs are involved in many biological and physical processes including pH homeostasis and have 16 mammalian isoforms (CA1 ~ CA 16) [28]. In PharmDB, TBZT is linked to carbonic anhydrase 2 (CA2). However, TBZT can suppress proliferation of lung cancer cell under hypoxic conditions only (Figure 5) and the expression of CA2 is not associated with hypoxic conditions. So we have extended cancer-linked CA isoforms in PharmDB (Figure 6A). As a result, we considered three different human CA isozymes (i.e., 1, 2, and 9) as targets of TBZT, and tested whether TBZT inhibits CA activity. TBZT suppressed all of the three CA isozymes with similar K_i_ values (Figure 6B). As a positive control, acetazolamide (AZA), a known inhibitor of carbonic anhydrases (CAs), was also used [29]. AZA also suppressed the activities of the three CAs, although the K_i_ values varied depending on the target enzymes. However, among the CA isozymes, CA9 is known to be induced in hypoxic conditions and has functional association with cancer [30]. Thus, the efficacy of TBZT against HCC-1588 cells is likely to have resulted from its inhibition of CA9. For that reasons, we decided to focus on CA9 as the major effective target of TBZT against cancer although we do not exclude the involvement of other isozymes.

CA9, a carbonic anhydrase isoenzyme, is a transmembrane protein that plays an important role in pH regulation [31]. The expression of CA9 is highly induced in various cancers under hypoxic conditions, which is functionally important for the growth and survival of tumor cells [31]. We confirmed whether CA9 is actually induced in hypoxic conditions by Western blotting with its specific antibody in HCC-1588. As expected, CA9 levels were significantly increased in hypoxic conditions (1% O_2_) compared with those in normoxic conditions (20% O_2_) (Figure 6C). We also confirmed that TBZT induced cell death by measuring the activation of caspase 3 (Figure 6D). To confirm the drug-protein pair relationship between CA9 and TBZT, we tested whether the forced expression of CA9 would compensate for the anti-proliferative activity of CA9 by the treatment of TBZT under hypoxic conditions. Cell proliferation was reduced to 70% of the control cells by the treatment of TBZT in the cells transfected with EV, but 35% of the control cells in the cells transfected with CA9. Therefore, the exogenous supplementation of CA9 recovered the proliferation by up to 35% (Figure 6E). This result validates that the anti-proliferative activity of TBZT against HCC-1588 cells mainly involves CA9. Perhaps, the remaining part could be contributed by other CA isozymes that are also involved in the regulation of cancer. Even if further chemical optimization of TBZT is required to improve efficacy and specificity, these results suggest a possible application of TBZT for further development against lung cancer through its CA9 inhibitory activity.

## Discussion

This study demonstrates that drug repositioning can be rapidly guided by a knowledge platform PharmDB, a pharmacological network database comprising protein, drug, and disease data which we are providing as a web-based service. As an ever-increasing amount of biological and pharmacological data are scattered throughout the literature and in proprietary databases, the integrated data of PharmDB provides a valuable tool by consolidating certain valuable sets of data. We adopted a tripartite pharmacological network-based analysis, and developed a novel neighborhood scoring algorithm to predict previously unknown relationships between drugs, proteins and diseases. The theoretical foundation of algorithm is that a connection probability between two nodes is proportional to the number of nodes commonly shared between them. So the connection probability of two indirectly linked nodes was computed, which is called the shared neighborhood score. This score can highlight missing linkages which may either result from “no actual connection” or “lack of information” and help to differentiate between these two possibilities.

We experimentally validated the usefulness of the shared neighborhood score by identifying a hitherto unknown drug-protein relationship and potential new indication based on this connection. Aside from drug repositioning, the network map of PharmDB composed of not only the data integrated from diverse databases but also the predicted data using the shared neighborhood algorithm can applied to other purposes, such as the prediction of drug mode-of-action, off-target effects, and even the design of optimal drug combinations for a disease of interest.

## Conclusions

PharmDB, an integrated tripartite database, coupled with Shared Neighborhood Scoring (SNS) algorithm, would provide much more enriched information than general integrated databases and give us clues for finding new indication of known drugs. Furthermore, these data can be easily explored with phExplorer and accessed via BioMart web service (http://www.i-pharm.org/, http://biomart.i-pharm.org/).

## Methods

### Construction of PharmDB

To integrate the data in the existing databases that contain different identifiers, we assigned the following standard identifiers (IDs): PubChem CID for drug, GeneID for protein, and MeSH descriptor for disease. We constructed a comprehensive drug-protein-disease tripartite network by integrating the link information from the nine databases, namely, EntrezGene interaction (ftp://ftp.ncbi.nih.gov/gene/GeneRIF/), MINT (ftp://mint.bio.uniroma2.it/pub/release/mitab26/2011-07-08/), CTD (http://ctd.mdibl.org/), TTD (http://bidd.nus.edu.sg/group/cjttd/TTD_Download.asp), ChemBank (http://chembank.broadinstitute.org), DIP (http://dip.doe-mbi.ucla.edu/dip/Download.cgi), PharmGKB (http://www.pharmgkb.org/resources/downloads_and_web_services.jsp), OMIM (ftp://ftp.ncbi.nih.gov/repository/OMIM/ARCHIVE/), and GAD (http://geneticassociationdb.nih.gov/). As the existing databases have their own unique ID systems, we tagged the standard IDs using an in-house script. For the entities that were not tagged by the in-house script, we manually assigned them with appropriate IDs.

### Shared neighborhood scoring algorithm

The shared neighborhood scoring algorithm is based on the basic principle that the connection probability of a link between two nodes (*i* and *j*) is roughly proportional to the number of nodes commonly shared between the original two nodes, *i* and *j* (Additional file 1: Figure S1). The shared neighborhood score $S_{\mathrm{ij}}$ is defined as $S_{\mathrm{ij}}=\sum_{k}W_{\mathrm{ik}}W_{\mathrm{kj}}$. In this equation, *i* and *j* indicate the indices of a pair of nodes; *k* is the index of a shared neighbor node; and $W_{\mathrm{ik}}$ is the weight of a link between *i* and *k*. The link between *i* (or *j*) and *k* can be real or virtual (i.e., having no known connection but is expected to be connected). Thus, we can define $W_{\mathrm{ik}}$ as $W_{\mathrm{ik}}=a_{\mathrm{ik}}+P\left(S_{\mathrm{ik}}^{\left(0\right)}\right)\delta_{a_{\mathrm{ik}},0}$. Here, $a_{\mathrm{ik}}=1$ and $\delta_{a_{ik^{0}}}=0$if the link between *i* and *k* is real; and $a_{\mathrm{ik}}=0$ and $\delta_{a_{\mathrm{ik}}}=1$ if the link is virtual. When there are only direct connections between node *i* and node *j*, a 0th-order shared neighborhood score $s_{\mathrm{ij}}^{\left(0\right)}$ becomes $S_{\mathrm{ij}}^{\left(0\right)}=\sum {}_{k}a_{\mathrm{ik}}a_{\mathrm{kj}}=n(0,1,2,\text{3…})$, where n is the number of bridging nodes between node *i* and node *j*. $P(S_{\mathrm{ik}}^{\left(0\right)})$ is a connection probability that depends on the value of the 0th-order shared neighborhood score $s_{\mathrm{ik}}^{\left(0\right)}$. For the 0th-order shared neighborhood score $s_{\mathrm{ik}}^{\left(0\right)}=n(=0,1,2,\ldots )$, the function P(n) is defined as follows:

(1) $$P\left(n\right)=\frac{\left(number\;of\;connected\;pairs\right)}{\left(number\;of\;pairs\right)}\;for\;n=1,2,\ldots$$

(2) P(n)=0forn=0

Based on the probability above, non-linear regression was carried out to extract connecting probability functions. Logistic function was used for this (See Additional file 1: Figure S2, Additional file 1: Table S1 for more details).

(3) $$f(x)=\frac{1}{1+e^{a+bx}}$$

The shared neighborhood score $s_{\mathrm{ij}}$ then becomes $s_{\mathrm{ij}}=s_{\mathrm{ij}}{}^{\left(0\right)}+s_{\mathrm{ij}}{}^{\left(1\right)}+s_{\mathrm{ij}}{}^{\left(2\right)}=\sum_{k}(a_{\mathrm{ik}}+P(s_{\mathrm{ik}}^{\left(0\right)})\delta_{a_{ik^{0}}})(a_{\mathrm{kj}}+P(s_{\mathrm{kj}}^{\left(0\right)})\delta_{a_{kj^{,0}}})$ where $s_{\mathrm{ij}}^{\left(0\right)}=\sum_{k}a_{\mathrm{ik}}a_{\mathrm{kj}}$,$s_{\mathrm{ij}}^{\left(1\right)}=\sum_{k}\left(a_{\mathrm{ik}}P(S_{\mathrm{kj}}{}^{\left(0\right)})\delta a_{kj^{,0}}+a_{\mathrm{kj}}P(S_{\mathrm{ik}}{}^{\left(0\right)})\delta_{a_{ik^{0}}}\right)$, and $s_{\mathrm{ij}}^{\left(2\right)}=P(s_{\mathrm{ik}}^{\left(0\right)})\delta_{a_{ik^{,0}}}P(s_{\mathrm{kj}}^{\left(0\right)})\delta_{a_{kj^{,0}}}$. Here, the 1st-order term $s_{\mathrm{ij}}^{\left(1\right)}$ is added when some nodes are linked directly to node *i* (or *j*) but linked indirectly to node *j* (or *i*). The 2nd-order term $s_{\mathrm{ij}}^{\left(2\right)}$ is considered only when some nodes are linked indirectly to both node *i* and node *j*. In Additional file 1: Figure S1, as node *m* is the only shared neighbor of node *i* and node *j*, $s_{\mathrm{ij}}^{\left(0\right)}=1$. To obtain the 1st-order shared neighborhood score $s_{\mathrm{ij}}^{\left(1\right)}$ or the 2nd-order shared neighborhood score $s_{\mathrm{ij}}^{\left(2\right)}$, connection probability $P(s_{\mathrm{ik}}^{\left(0\right)})$ is calculated beforehand. As a pair (*i*, *k*) is mediated by two nodes, $W_{\mathrm{ik}}=P(2)$, Path (*i*, *k*, *j*) is composed of an indirect link ( *i*, *k*) and a direct link ( *k*, *j*). Similarly, $W_{\mathrm{il}}=P(3)$ and $W_{\mathrm{lj}}=P(1)$. Path (*i*, *l*, *j*) is composed of both indirect links ( *i*, *l*) and ( *l*, *j*). The total shared neighborhood score is thus $s_{\mathrm{ij}}=s_{\mathrm{ij}}^{\left(0\right)}+s_{\mathrm{ij}}^{\left(1\right)}+s_{\mathrm{ij}}^{\left(2\right)}=1+P(2)+P(3)P(1)$. When calculating $s_{\mathrm{ij}}^{\left(2\right)}$, we omitted a link between node *i* and *j* to remove the dependency of the measure on the existence of a link between *i* and *j,* which is the so-called “leave-one-out approach” [32].

The shared neighborhood score is proportional to the number of shared neighborhood nodes. So there is no an upper limit on score. The problem is that the amount of data is not evenly distributed on each relation category. So, even if two different types of relations have identical score, their connecting possibility can’t be regarded as identical. For that reason, we normalized the shared neighborhood score using connecting probability function (Additional file 1: Figure S3).

### FDA approved drugs

We have downloaded Drugs@FDA data files (Last updated: 19/09/2011)(http://www.fda.gov/downloads/Drugs/InformationOnDrugs/UCM163762). Then we extracted single active ingredient from Product table and tagged PubChem ID for them. The total number of FDA approved drugs tagged with PubChem ID is 23,191.

### Cell culture and materials

The HCC-1588 cell line was obtained from the Korean cell line bank and was maintained in RPMI (Hyclone) containing 10% fetal bovine serum and 1% antibiotics. Antibody against caspase-3 and tublin (Cell Signaling Technology) were purchased. M73 monoclonal antibody to CA9 was obtained from Dr. S. Pastorekova (Slovak Academy of Science, Slovak Republic). TBZT and AZA were purchased from Sigma.

### Thymidine incorporation assay

To determine the effect of TBZT on cell proliferation, HCC-1588 cells were treated with TBZT in 2% serum-containing media for 48 h under normoxic (20% O_2_) and hypoxic (1% O_2_) conditions. AZA was used as positive control. pcDNA3-CA9 vector and empty vector (Dr. J.-Y. Kim, National Cancer Center, Korea) were transfected into HCC-1588 cells using Lipofectamine 2000 (Invitrogen). After 24 h incubation, TBZT was added to 2% serum-containing media for 48 h under hypoxic conditions. [^3^ H] thymidine at 1 ***μ***Ci/ml was added to the culture medium and was incubated for 4 h. The incorporated thymidine was measured by liquid scintillation counter (Wallac).

### Flow cytometry

HCC-1588 cells were treated with TBZT (0.4, 2, 10 μM) in 2 % serum-containing medium for 48 h under normoxic and hypoxic conditions. AZA was used as positive control. The treated cells were fixed with 70% ethanol for 1 h at 4°C, washed twice with ice-cold PBS, and stained with propidium iodide (50 μg/ml) containing 0.1% sodium citrate, 0.3% NP-40 (nonylphenoxylpolyethoxylethanol 40), and 50 μg/ml RNase A for 40 min. The cells were subjected to flow cytometry (FACSCalibur, Becton-Dickinson) to evaluate the apoptotic cells by counting the sub-G1 cells. For each sample, 20,000 cells were analyzed using Cell Quest Pro software.

### Enzyme activity

An applied photophysics stopped-flow instrument was used for assaying CA-catalyzed CO_2_ hydration activity [33]. Following the initial rates of the CA-catalyzed CO_2_ hydration reaction for a period of 10–100 s, phenol red (at a concentration of 0.2 mM) was used as the indicator, working at the absorbance maximum of 557 nm in 20 mM HEPES buffer (pH 7.5) and 20 mM Na_2_SO_4_ (to maintain the constant ionic strength). For the determination of the kinetic parameters and inhibition constants, the CO_2_ concentrations used ranged from 1.7–17 mM. For each inhibitor, at least six traces of the initial 5–10% of the reaction were used for determining the initial velocity. The uncatalyzed rates were determined in the same manner and were subtracted from the total observed rates. Stock solutions of the inhibitor (0.1 mM) were prepared in distilled-deionized water and diluted to 0.01 nM with distilled-deionized water. Inhibitor and enzyme solutions were preincubated together for 15 min–72 h at room temperature (15 min) or 4°C (all other incubation times) prior to assay to allow the formation of the enzyme-inhibitor complex or the eventual active site mediated hydrolysis of the inhibitor. The inhibition constants were obtained by non-linear least-squares methods using PRISM 3 as previously described. The mean values were represented from at least three different determinations [31,34].

## Abbreviations

HTS, Highthrouput Screening; SNS, Shared Neighborhood Scoring; SN score, Shared Neighborhood score; MINT, the Molecular INTeraction database; DIP, the Database of Interacting Proteins; CTD, The Comparative Toxicogenomics Database; TTD, Therapeutic Target Database ChemBank; PharmGKB, The Pharmacogenomics Knowledge Base; OMIM, Online Mendelian Inheritance in Man; GAD, Genetic Association Database; ROC, Receiver Operating Characteristic; AUC, Area Under Curve; AZA, Acetazolamide; CA, Carbonic anhydrase; SCC, Squamous Cell Carcinoma; TBZT, Thia-benzthiazide; KS test, Kolmogorov-Smirnov test.

## Competing Interests

The authors declare that they have no competing interests.

## Authors’ contributions

KR, LC and SK conceived the ideas for this study. KR, JHL, TB and HSL designed and performed the computational systems biology approach. KR, JHL and TB designed and constructed the database. KR and TB developed the algorithm. HSL and JHL designed and performed the repositioning pipeline. HSL, DGK and YSO performed the cell biology experiments. AI and CTS performed the enzyme activity analysis. SK wrote the manuscript with contributions from all the participating authors.

## Authors’ information

Current positions: KR, YJ - Korean Bioinformation Center (KOBIC), Korea Research Institute of Bioscience and Biotechnology (KRIBB), Daejeon 305–806, Korea

## Supplementary Material

Additional file 1 **Supplementary figures and tables.** Detailed Description of Shared neighborhood score algorithm and algorithm Validation. Detailed description of the algorithm including non-linear regression results for extracting connecting probability functions. And a list of inferred SCC drug candidates is included.References. Click here for file
