# Supplementary material for: Rational drug repositioning guided by an integrated pharmacological network of protein, disease and drug
Source: BMC Syst Biol. 2012 Jul 2;6:80. doi: 10.1186/1752-0509-6-80 (PMC3443412; doi:10.1186/1752-0509-6-80)
Supplement: Additional file 1 — Supplementary figures and tables. Detailed Description of Shared neighborhood score algorithm and algorithm Validation. Detailed description of the algorithm including non-linear regression results for extracting connecting probability functions. And a list of inferred SCC drug candidates is included.References. [file 1752-0509-6-80-S1.pdf]

Figure S1. Design of shared neighborhood score algorithm

SN Score = $f_{(Share\_Count + Share\_Weight)}$

Share weight = Sum of connecting probability

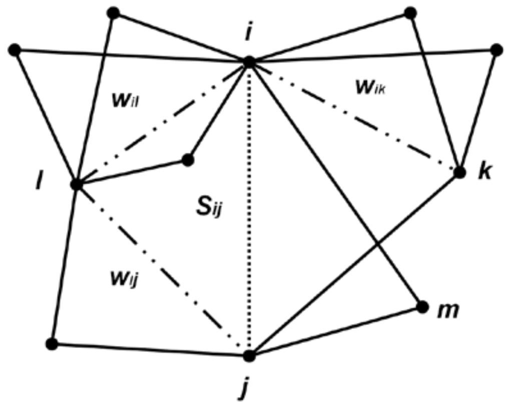

Share weight

$$S_{ij} = S_{ij}^{(0)} + \underbrace{S_{ij}^{(1)} + S_{ij}^{(2)}}_{\text{Share weight}}$$

$$= \sum_k (a_{ik} + P(S_{ik}^{(0)})\delta_{a_{ik},0})(a_{kj} + P(S_{kj}^{(0)})\delta_{a_{kj},0})$$

where,

$S_{ij}^{(0)} = \sum_k a_{ik} a_{kj}$

$S_{ij}^{(1)} = \sum_k (a_{ik} P(S_{kj}^{(0)})\delta_{a_{kj},0} + a_{kj} P(S_{ik}^{(0)})\delta_{a_{ik},0})$

$S_{ij}^{(2)} = \sum_k P(S_{ik}^{(0)})\delta_{a_{ik},0} P(S_{kj}^{(0)})\delta_{a_{kj},0})$

a_{ik}=1 and $\delta_{a_{ik},0}$=0 if the link between i and k is real

a_{ik}=0 and $\delta_{a_{ik},0}$=1 if the link is virtual

$P(x)$ = connecting probability function

$$P_{(x)} = \frac{(\text{number of connected pairs})}{(\text{number of pairs})} \text{ for n} = 1,2,\ldots$$

$$P_{(x)} = 0 \text{ for } x = 0$$

Logistic function is used for non-linear regression.

$$f(x) = \frac{1}{1 + e^{a+bx}}$$

Figure S2. Non-linear regression results for extracting connecting probability function

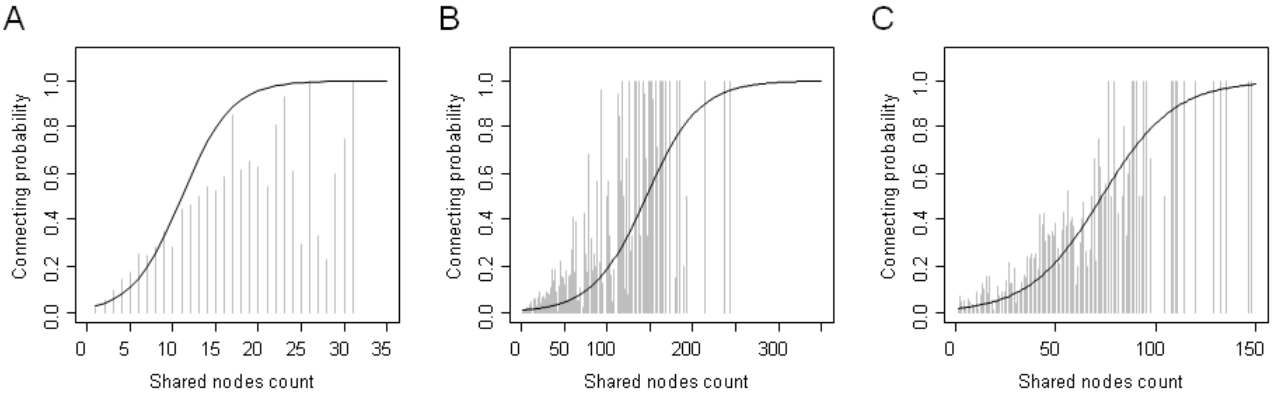

Non-linear connecting probability model based on the number of nodes commonly shared between two nodes. (A) Drug-Protein. (B) Drug-Disease. (C) Protein-Disease.

Table S1. Model coefficient values from non-linear regression for connecting probability function

Model Coefficient	Drug-Protein	Drug-Disease	Protein-Disease
a	3.87	4.67	4.03
b	-0.35	-0.03	-0.06

Figure S3. Connecting probability model for SN score

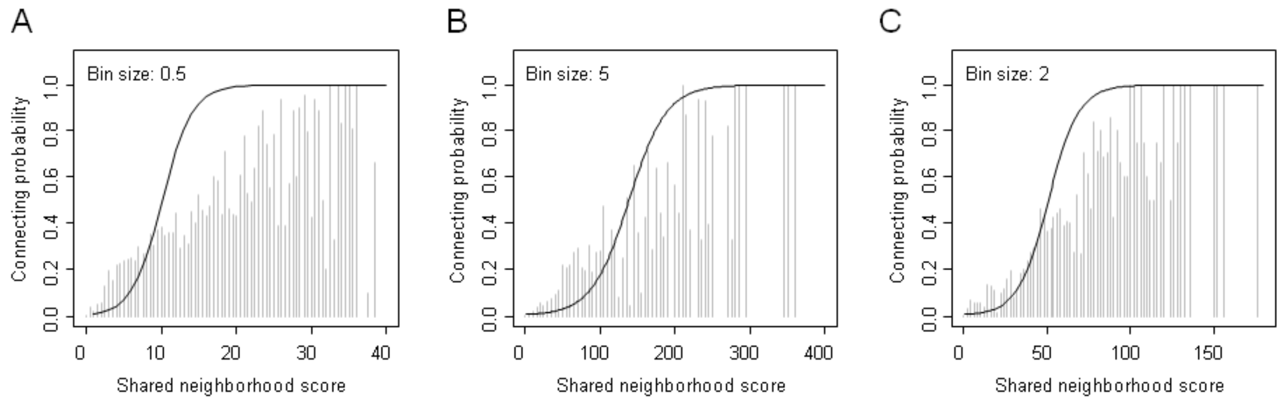

The shared neighborhood score is proportional to the number of shared neighborhood nodes. As the amount of data is not evenly distributed on each relation category, even if two different types of relations have identical score, their connecting possibility can't be regarded as identical. Therefore the shared neighborhood score is normalized using connecting probability function. (A) Drug-Protein. (B) Drug-Disease. (C) Protein-Disease.

Table S2. Model coefficient values from connecting probability model for SN score

Model Coefficient	Drug-Protein	Drug-Disease	Protein-Disease
a	5.09	5.62	5.53
b	-0.50	-0.11	-0.04

Table S3. Inferred SCC drug candidates

Twenty eight SCC drug candidates which satisfy four criteria; 1) inferred by SNS algorithm 2) FDA approved drugs 3) non-cancer drugs 4) directly linked to cancer-related proteins.

Name	Cancer-related GeneID	Cancer-related GeneSymbol
MIRTAZAPINE	3157	HMGCS1
ZONISAMIDE	7364	UGT2B7
ARGATROBAN	2147	F2
GANCICLOVIR	6580	SLC22A1
ACYCLOVIR	6580	SLC22A1
CLADRIBINE	9154	SLC28A1
BENZTHIAZIDE	760	CA2
HYDROCHLOROTHIAZIDE	760	CA2
CHLOROTHIAZIDE	760	CA2
DIAZOXIDE	3767	KCNJ11
DROPERIDOL	3757	KCNH2
FLURBIPROFEN	8856	NR1I2
GLIMEPIRIDE	8856	NR1I2
GLIPIZIDE	1559	CYP2C9
METHYLDOPA	1312	COMT
MEPROBAMATE	5581	PRKCE
MYCOPHENOLATE MOFETIL	54576	UGT1A8
AZITHROMYCIN	1813	DRD2
PINDOLOL	155	ADRB3
FLUPHENAZINE DECANOATE	3351	HTR1B
PROCHLORPERAZINE	1813	DRD2
LATANOPROST	5737	PTGFR
NALOXONE	8856	NR1I2
NALBUPHINE	4985	OPRD1
CABERGOLINE	1813	DRD2
TOBRAMYCIN	4549	RNR1
ZAFIRLUKAST	10800	CYSLTR1
NATEGLINIDE	1559	CYP2C9
